# Synthesis and Photophysical Study of 2′-Deoxyuridines Labeled with Fluorene Derivatives

**DOI:** 10.3390/molecules171012061

**Published:** 2012-10-15

**Authors:** Hyun Yi Cho, Sang Keun Woo, Gil Tae Hwang

**Affiliations:** 1 Department of Chemistry and Green-Nano Materials Research Center, Kyungpook National University, Daegu 702-701, Korea; Email: whgusdl840@hanmail.net; 2 Molecular Imaging Research Center, Korea Institute of Radiological and Medical Sciences, 75 Nowon-gil, Seoul 139-706, Korea

**Keywords:** nucleosides, fluorene, fluorescence, dibenzofuran, dibenzothiophene

## Abstract

We examined microenvironment-sensitive fluorescent 2′-deoxyuridines labeled with fluorene derivatives that exhibited solvent-dependent photophysical properties. The high sensitivity of the fluorescence shift and the nucleoside intensity dependence on solvent polarity provided information useful for estimating the polarity of the environment surrounding the fluorescent nucleoside.

## 1. Introduction

Fluorescent nucleosides which are structurally noninvasive, forming stable Watson-Crick base pairs, and sensitive to their physical conditions and molecular species in solution, exhibiting environmental-specific changes in their fluorescent properties, have become powerful tools for the investigation of nucleic acid structure, recognition of single nucleotide polymorphisms (SNPs), and studies on enzymatic processes involving DNA [1,2,3,4,5,6,7,8].

**Figure 1:**
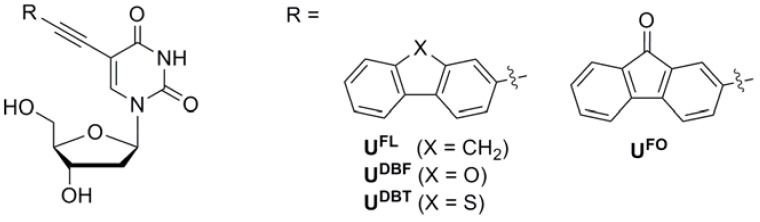
Fluorescent nucleosides used in this study.

In order to design fluorescent nucleosides, we utilized an ethynyl linker at the 5 position of uracil to maintain the hybridization properties of the parent nucleoside. This substitution is expected to have very little influence on the stability of the resulting duplex DNA [9,10,11,12,13,14,15,16,17,18,19,20]. Among fluorophores, fluorene derivatives have moderate quantum yields and are less bulky than other commonly used fluorophores, e.g., pyrene, fluorescein, rhodamine, and cyanine dyes [21]. Previously, we reported fluorene (FL)- and 9-fluorenone (FO)-labeled deoxyuridine (**U^FL^** and **U^FO^**), which we incorporated at the central positions of oligodeoxynucleotides in an attempt to examine the effect of electronic modification of the fluorophore scaffold on the potential of the molecular beacon (MB) for SNP typing (Figure 1) [9,10,11]. When such a quencher-free MB hybridizes with its perfectly matched target DNA, it exhibits strong fluorescence. In contrast, when it forms duplexes with single-base-mismatched target DNAs, the **U^FL^** and **U^FO^** units display quenched fluorescence as a result of photoinduced charge transfer originating from interactions with neighboring nucleobases. These changes in fluorescence are extremely dependent on the electronic and conformational microenvironments of the flanking bases. Therefore, we sought to synthesize other fluorescent uridines labeled with new FL derivatives, dibenzofuran (DBF) and dibenzothiophene (DBT), in order to examine changes in their photophysical properties through modifications of the fluorene unit and to develop these nucleosides as microenvironment-sensitive fluorescent nucleosides [17,22,23]. Although FL, FO, DBF, and DBT are structural analogs that differ only in the type of atoms bridging the two aromatic rings, they have dramatically different photophysical properties [24,25,26]. Here, we report the synthesis and photophysical properties of fluorescent FL derivative-conjugated 2′-deoxyuridine analogs.

## 2. Results and Discussion

**Scheme 1:**
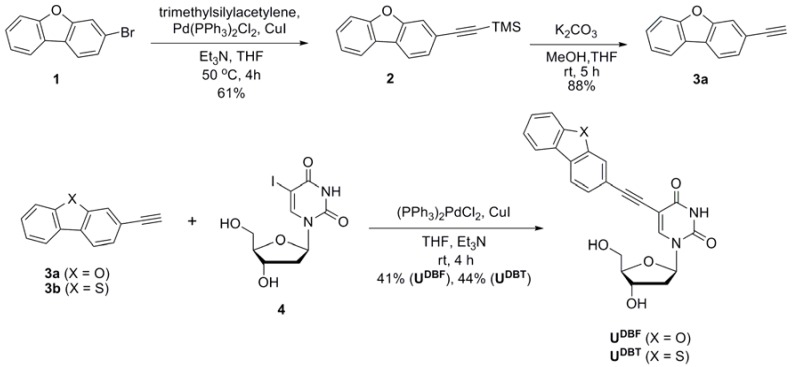
Route for the synthesis of **U^DBF^** and **U^DBT^**.

The synthetic route of the DBF- and DBT-labeled 2′-deoxyuridine derivatives **U^DBF^** and **U^DBT^** is outlined in Scheme 1. 3-Ethynyldibenzofuran (**3a**) was prepared by Pd/Cu-catalyzed Sonogashira coupling [27,28] of 3-bromodibenzofuran (**1**) with trimethylsilylacetylene followed by desilylation. 3-Ethynyldibenzothiophene (**3b**) was also synthesized according to the reported protocol [29]. We synthesized **U^DBF^** and **U^DBT^** from the corresponding 2′-deoxy-5-iodouridine (**4**) through a palladium catalyzed cross-coupling reaction with 3-ethynyldibenzofuran (**3a**) or 3-ethynyldibenzothiophene (**3b**). The syntheses of **U^FL^** and **U^FO^** were conducted as reported [9,10,11].

**Figure 2:**
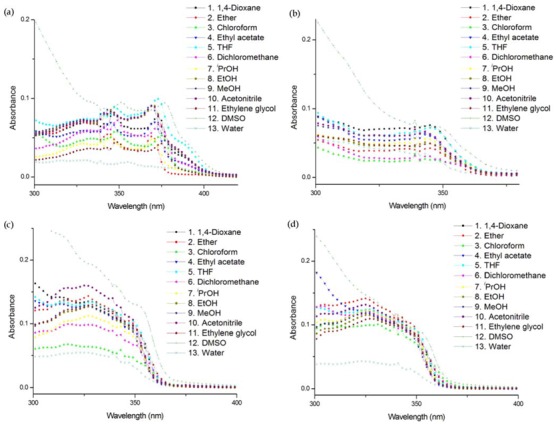
Absorption spectra of (**a**) **U^FL^** (3 μM), (**b**) **U^FO^** (3 μM), (**c**) **U^DBF^** (5 μM), and (**d**) **U^DBT^** (5 μM) in different solvents at 25°C. All samples contain 0.5% THF/MeOH (1:1 v/v) to ensure solubility.

**Figure 3:**
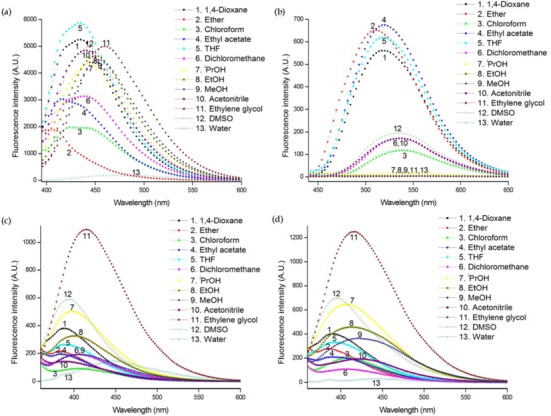
Emission spectra of (**a**) **U^FL^**, (**b**) **U^FO^**, (**c**) **U^DBF^**, and (**d**) **U^DBT^** in different solvent at 25°C (all at 3 μM concentration). The excitation wavelengths were 370 nm for **U^FL^** and 340 nm for the others. All samples contain 0.5% THF/MeOH (1:1 v/v) to ensure solubility.

Generally, solvent polarity is of primary interest when considering environmental effects [30]. Therefore, we first measured the absorption and emission spectra of nucleosides in thirteen solvents of different polarities. Solvent marginally affected the absorption, probably due to the weak interaction between the nucleosides and solvent in the ground state (Figure 2). However, solvent polarity had a significant influence on both the emission maximum and intensity (Figure 3). All nucleosides exhibited different emission intensities and maxima depending on the solvent they were in, indicating that they are all environmentally sensitive.

Table 1 summarizes the photophysical properties of nucleosides in thirteen different solvents. The fluorescence quantum yields (*Φ*_F_) of the nucleosides were determined using a 0.1 N aqueous H_2_SO_4_ solution of quinine sulfate (*λ*_ex_ = 350 nm) as a standard [31]. There are some noteworthy features: (a) generally, the presence of a heteroatom in the fluorene unit of nucleoside **U^FO^**, **U^DBF^**, and **U^DBT^** diminishes its fluorescence yield and fluorescence brightness (*i.e.*, the product of its molar extinction coefficient and quantum yield) drastically when compared with **U^FL^**. (2) **U^DBF^** and **U^DBT^** showed very similar photophysical properties in various solvents. (3) The quantum yield and fluorescence brightness of nucleosides is highest in *^i^*PrOH for **U^FL^**, ethyl acetate for **U^FO^**, and ethylene glycol for **U^DBF^** and **U^DBT^**. The lowest fluorescence brightness, however, was observed in ethylene glycol for **U^FO^** and water for **U^FL^**, **U^DBF^**, and **U^DBT^**. These results indicate that the nucleosides exhibit highly solvent-dependent photophysical properties despite their structural similarities. **U^FO^**, interestingly, exhibited a strong solvent dependency–namely, higher fluorescence brightness in aprotic solvents relative to those in protic solvents such as *^i^*PrOH, EtOH, MeOH, ethylene glycol, and water which was attributable to the hydrogen bonding between the carbonyl group of **U^FO^** and solvent. 

**Table 1:** Photophysical characteristics of nucleosides in different solvents at 25°C.

Solvent	Compound	*E*_T_(30)^11^	*λ*_max_ (nm) ^a^	ε (M^−1^ cm^−1^)	*λ*_em_ (nm) ^b^	*Φ*_F_^c^	Brightness ^d^
1,4-Dioxane	**U^FL^**	36	373	25,000	434	0.33	8,250
Ether		34.5	370	27,200	409	0.055	1,500
Chloroform		39.1	375	20,100	420	0.18	3,620
Ethyl acetate		38.1	371	19,900	414	0.14	2,790
THF		37.4	373	29,700	434	0.31	9,200
Dichloromethane		40.7	374	22,200	439	0.23	5,100
*^i^*PrOH		48.4	370	20,900	444	0.50	10,500
EtOH		51.9	370	24,400	450	0.25	6,100
MeOH		55.4	369	25,100	453	0.26	6,530
Acetonitrile		45.6	370	26,200	440	0.28	7,340
Ethylene glycol		56.3	374	12,900	460	0.27	3,480
DMSO		45.1	377	27,100	443	0.18	4,880
Water		63.1	384	4,930	467	0.062	305
1,4-Dioxane	**U^FO^**	36	344	20,800	518	0.056	1,160
Ether		34.5	345	21,200	511	0.080	1,700
Chloroform		39.1	343	14,300	538	0.029	415
Ethyl acetate		38.1	343	19,400	519	0.090	1,750
THF		37.4	346	21,900	519	0.064	1,400
Dichloromethane		40.7	342	15,800	535	0.040	632
*^i^*PrOH		48.4	344	16,500	552	0.0034	56.1
EtOH		51.9	343	16,300	554	0.00097	15.8
MeOH		55.4	342	17,900	558	0.0014	25.1
Acetonitrile		45.6	342	18,400	537	0.0219	403
Ethylene glycol		56.3	345	14,000	556	0.00076	10.6
DMSO		45.1	347	21,500	535	0.018	387
Water		63.1	341	13,200	552	0.0038	50.2
1,4-Dioxane	**U^DBF^**	36	328	23,300	388	0.049	1,140
Ether		34.5	327	27,300	383	0.029	792
Chloroform		39.1	317	17,100	404	0.086	1,470
Ethyl acetate		38.1	327	24,500	383	0.027	662
THF		37.4	329	24,700	388	0.035	865
Dichloromethane		40.7	328	21,400	405	0.044	942
*^i^*PrOH		48.4	328	22,500	397	0.086	1,940
EtOH		51.9	327	24,300	401	0.074	1,800
MeOH		55.4	326	23,000	406	0.047	1,080
Acetonitrile		45.6	326	24,000	386	0.026	624
Ethylene glycol		56.3	329	21,300	415	0.23	4,900
DMSO		45.1	nd ^e^	nd ^e^	394	0.084	nd ^e^
Water		63.1	325	11,700	449	0.047	550
1,4-Dioxane	**U^DBT^**	36	327	26,500	390	0.047	1,250
Ether		34.5	325	28,900	358	0.020	578
Chloroform		39.1	329	20,500	407	0.050	1,030
1,4-Dioxane	**U^DBT^**	36	327	26,500	390	0.047	1,250
Ethyl acetate		38.1	325	24,200	389	0.024	581
THF		37.4	326	24,900	391	0.033	822
Dichloromethane		40.7	327	24,600	408	0.029	713
*^i^*PrOH		48.4	325	24,300	407	0.064	1,560
EtOH		51.9	326	22,900	411	0.061	1,400
MeOH		55.4	325	24,400	421	0.045	1,100
Acetonitrile		45.6	325	24,800	423	0.020	496
Ethylene glycol		56.3	328	21,900	417	0.11	2,410
DMSO		45.1	nd ^e^	nd ^e^	395	0.082	nd ^e^
Water		63.1	324	8,600	451	0.0079	67.9

^a^ Only the largest absorption maxima are listed; ^b^ Wavelength of emission maximum when excited at the absorption maximum; ^c^ Quantum efficiencies using 0.1 N aqueous H_2_SO_4_ solution of quinine sulfate as a standard, *λ*_ex_ = 350 nm. Data shown are the mean values of three independent experiments; ^d^ The fluorescence brightness = *ε* × *Φ*_F_; ^e^ Not detectable due to overlapping absorption bands of a nucleoside and DMSO.

**Figure 4:**
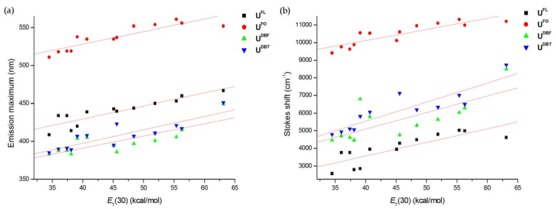
Effect of *E*_T_(30) on (**a**) the fluorescence emission maxima and (**b**) the Stokes shifts of nucleosides.

In polar solvents such as *^i^*PrOH, EtOH, MeOH, acetonitrile, ethylene glycol, DMSO, and water substantially larger red-shifts in emission maxima of nucleosides were observed. Because it is instructive to calculate the magnitude of the expected spectral shifts due to solvent polarity effects, we plotted the fluorescence emission maxima and Stokes shifts (*ν*_abs_–*ν*_em_) of nucleosides in thirteen different solvents against Reichardt’s microscopic solvent parameter, *E*_T_(30) (Figure 4) [32]. It is interesting to note that there is a linear correlation between emission maxima and *E*_T_(30) regardless of the aproticity of the solvent. The red-shift of the fluorescence could be due to the significant difference between the excited‐state charge distribution in the solute and the ground‐state charge distribution, resulting in stronger interactions with polar solvents in the excited state. 

**Figure 5:**
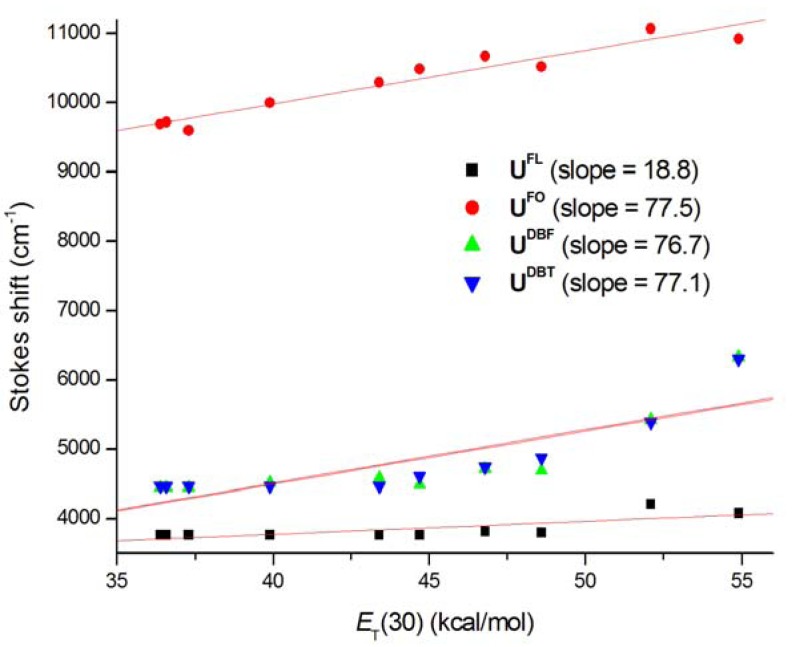
Dependence of the Stokes shift of nucleosides in water/1,4-dioxane binary solvent mixture on the empirical solvent polarity parameter, *E*_T_(30).

Emission maxima of **U^FO^** were red-shifted relative to those of other nucleosides. This higher Stokes shift of **U^FO^** is probably because the carbonyl group allows for hydrogen bonding and charge separation better than do the other nucleosides [30]. Interestingly, the Stokes shifts of **U^DBF^** and **U^DBT^** exhibited a more gradual shift to longer wavelengths with increasing solvent polarity compared to the slopes of other nucleosides, as shown in Figure 4b. In order to compare the sensitivity of our molecules of interest to environmental polarity with that of reported polarity-sensitive nucleosides [12,33], we examined the photophysical properties of fluorescent nucleosides in binary water/1,4-dioxane mixtures (Table S1, Figure 5), which is an established method for estimating the microenvironment polarity of fluorophores [34]. The Stokes shifts plotted against the *E*_T_(30) values of the samples is shown in Figure 5. The slopes obtained from the linear plots indicated that **U^FO^**, **U^DBF^**, and **U^DBT^** are highly sensitive to environmental polarity and are comparable to the slopes of reported nucleosides such as pyridine- and furan-labeled uridines. **U^DBF^** and **U^DBT^** revealed a seemingly exponential trend, leading us to conclude that a more appropriate expression for the interactions between these nucleosides and solvents should be explored.

## 3. Experimental

### 3.1. General

All reactions were performed in dry glassware under Ar atmospheres. Analytical thin layer chromatography (TLC) was performed using Merck 60 F_254_ silica gel plates; column chromatography was performed using Merck 60 silica gel (230–400 mesh). Melting points were determined using an Electrothermal IA 9000 series melting point apparatus and are uncorrected. Infrared (IR) spectra were recorded using a JASCO FT/IR-4100 spectrometer. ^1^H- and ^13^C-NMR spectra were recorded using a Bruker NMR spectrometer (AVANCE digital 400 MHz). High-resolution electron impact (EI) mass spectra were recorded using a JEOL JMS-700 mass spectrometer at the Daegu center of KBSI, Korea.

### 3.2. Materials

All commercially available chemicals were used without further purification; solvents were carefully dried and distilled prior to use. 3-Bromobenzofuran (**1**) [35] and 3-ethynyldibenzothiophene (**3b**) [29] have been reported previously. **U^FL^** and **U^FO^** were synthesized according to the reported protocol [9,10,11].

### 3.3. Preparation of 3-[2-(Trimethylsilyl)ethynyl]dibenzofuran (**2**)

A solution of **1 [35]** (580 mg, 2.35 mmol), (PPh_3_)_2_PdCl_2_ (165 mg, 0.235 mmol), and CuI (44.8 mg, 0.235 mmol) in THF (12 mL) and Et_3_N (3.9 mL) was degassed with nitrogen. Trimethylsilylacetylene (500 μL, 3.52 mmol) was added at 50 °C and the mixture stirred for 4 h. After evaporation of solvent *in vacuo*, the residue was subjected to chromatography on a silica gel column with hexane as eluent to give **2** (380 mg, 61%): M.p. 110–113 °C; IR (film): *ν* 3063, 2954, 2896, 2144, 1453, 1416, 1340, 1315, 1248, 1201, 1133, 940, 831, 743, 629 cm^–1^; ^1^H-NMR (CDCl_3_): *δ* 7.93 (dq, *J *= 8.0, 0.67 Hz, 1H; H-6), 7.86 (dd, *J *= 8.0, 0.40 Hz, 1H; H-1), 7.67 (q, *J *= 0.80 Hz, 1H; H-4), 7.57 (dt, *J *= 8.0, 0.80 Hz, 1H; H-2), 7.49–7.45 (m, 2H; H-7 and H-9), 7.35 (td, *J *= 7.4, 0.80 Hz, 1H; H-8), 0.29 (s, 9H; SiCH_3_); ^13^C-NMR (CDCl_3_): *δ* 156.9, 155.7 127.8, 127.1, 124.8, 123.9, 123.1, 121.8, 121.0, 120.5, 115.3, 111.9, 105.3, 95.0, 0.1; HRMS–EI (*m*/*z*): [M]^+^ calcd for C_1__7_H_16_OSi 264.0970; found, 264.0968.

### 3.4. Preparation of 3-Ethynyldibenzofuran (**3a**)

A solution of **2** (600 mg, 2.27 mmol) and K_2_CO_3_ (345 mg, 2.25 mmol) in MeOH (6.7 mL) and THF (6.7 mL) was stirred at rt for 5 h. After evaporation of the solvent *in vacuo*, dichloromethane and water were added and the product was extracted into the organic phase which was then concentrated. The residue was purified by chromatography (SiO_2_; hexane/EtOAc, 10:1) to give **3a** (385 mg, 88%): M.p. 83–86 °C; IR (film): *ν* 3264, 2920, 2854, 2098, 1641, 1595, 1446, 1364, 1193, 1107, 926, 880, 821, 742, 666, 606 cm^–1^; ^1^H-NMR (CDCl_3_): *δ* 7.94 (dq, *J *= 7.4, 0.8 Hz, 1H; H-6), 7.89 (dd, *J *= 8.0, 0.8 Hz, 1H; H-1), 7.70 (q, *J *= 0.53 Hz, 1H; H-4), 7.58 (dt, *J *= 8.0, 0.8 Hz, 1H; H-2), 7.50–7.46 (m, 2H: H-7 and H-9), 7.36 (td, *J *= 7.4, 0.80 Hz, 1H; H-8), 3.17 (s, 1H; CCH); ^13^C-NMR (CDCl_3_): *δ *156.9, 155.7, 127.9, 127.1, 125.1, 123.8, 123.2, 121.1, 120.7, 120.6, 115.5, 111.6, 83.9, 77.8; HRMS–EI (*m*/*z*): [M]^+^ calcd for C_14_H_8_O 192.0575; found, 192.0573.

### 3.5. General Procedure for Nucleoside Synthesis

(PPh_3_)_2_PdCl_2_ (36.5 mg, 0.0520 mmol) and CuI (9.9 mg, 0.0520 mmol) were added to a solution of 2′-deoxy-5-iodouridine **2** (184 mg, 0.520 mmol) and 2-ethynylfluorene derivative **3** (0.520 mmol) in Et_3_N (2.6 mL) and THF (7.8 mL). Argon was bubbled through the mixture for 2 min before the mixture was subjected 10 times to a pump/purge cycle, and then it was stirred at rt for 4 h. After evaporation of solvent in vacuo, the residue was subjected to chromatography (SiO_2_; CH_2_Cl_2_/MeOH, 40:1) to yield **U^DBF^** (41%) or **U^DBF^** (44%).

*2′-Deoxy-5-(3-dibenzofuranylethynyl)uridine* (**U^DBF^**). M.p. >164 °C dec.; IR (film): *ν* 3383, 3162, 3049, 2922, 2855, 1664, 1455, 1275, 1195, 1099, 987, 860, 740, 633 cm^–1^; ^1^H-NMR (DMSO-*d*_6_): *δ* 11.74 (s, 1H; NH), 8.46 (s, 1H; H-6), 8.18 (dd, *J *= 7.8, 0.60 Hz, 2H; DBF-H), 7.81 (q, *J *= 0.67 Hz, 1H; DBF-H), 7.74–7.72 (m, 1H; DBF-H), 7.58–7.54 (m, 1H; DBF-H), 7.51 (dd, *J *= 7.8, 1.4 Hz, 2 H; DBF-H), 7.43 (td, *J *= 7.3, 0.6 Hz, 1H; DBF-H), 6.15 (t, *J *= 6.4 Hz, 1H; H-1′), 5.30 (d, *J *= 4.4 Hz, 1H; OH-3′), 5.23 (t, *J *= 4.8 Hz, 1H; OH-5′), 4.30–4.26 (m, 1H; H-3′), 3.83 (q, *J *= 3.3 Hz, 1H; H-4′), 3.71–3.59 (m, 2H; H-5′), 2.20–2.17 (m, 2H; H-2′); ^13^C-NMR (DMSO-*d*_6_): *δ *161.5, 156.1, 155.1, 149.5, 144.2, 131.6, 128.3, 126.5, 124.0, 123.5, 123.1, 121.6, 121.3, 114.3, 111.8, 98.1, 92.0, 87.6, 84.9, 83.3, 69.9, 60.8; HRMS–EI (*m*/*z*): [M]^+^ calcd for C_23_H_18_N_2_O_6_, 418.1165; found, 418.1167.

*2'-Deoxy-5-(3-dibenzothiophenylethynyl)uridine* (**U^DB^^T^**). M.p. >165°C dec.; IR (film): *ν* 3377, 3155, 3053, 2923, 2852, 1660, 1455, 1272, 1228, 1195, 1094, 987, 919, 825, 747, 635 cm^–1^; ^1^H-NMR (DMSO-*d*_6_): *δ* 11.72 (s, 1H; NH), 8.46 (s, 1H; H-6), 8.40–8.38 (m, 2H; DBT-H), 8.178 (dd, *J *= 1.4, 0.60 Hz, 1H; DBT-H), 8.07–8.04 (m, 1H; DBT-H), 7.58 (dd, *J *= 8.2, 1.4 Hz, 1H; DBT-H), 7.55–7.53 (m, 2H; DBT-H), 6.144 (t, *J *= 6.402, 1H; H-1′), 5.30 (d, *J *= 4.4 Hz, 1H; OH-3′), 5.23 (t, *J *= 4.6 Hz, 1H; OH-5′), 4.30–4.26 (m, 1H; H-3′), 3.83 (q, *J *= 3.3 Hz, 1H; H-4′), 3.71–3.59 (m, 2H; H-5′), 2.20–2.16 (m, 2H; H-2′); ^13^C-NMR (DMSO-*d*_6_): *δ* 161.6, 149.6, 144.2, 139.3, 138.9, 135.0, 134.5, 127.6, 125.7, 125.0, 123.2, 122.4, 122.2, 120.9, 98.1, 91.9, 87.6, 84.9, 83.5, 79.2, 69.9, 60.8, 55.0; HRMS-EI (*m*/*z*): [M]^+^ calcd for C_23_H_18_N_2_O_5_S, 434.0936; found, 434.0935.

### 3.6. UV and Fluorescence Measurements

Ultraviolet (UV) spectra were recorded using a Cary 100 UV-Vis spectrophotometer and 10-mm-path quartz cell, with respect to a pure-solvent reference. Fluorescence spectra were recorded using a Hitachi F4500 spectrofluorometer. All samples were prepared from a stock solution in THF/MeOH (1:1 v/v) to ensure solubility, and hence, all samples contain 0.5% THF/MeOH (1:1 v/v). The excitation and emission bandwidth was 1 nm. The fluorescence quantum yields (*Φ*_F_) were determined using 0.1 N aqueous H_2_SO_4_ solution of quinine sulfate as a reference [31].

## 4. Conclusions

We designed structurally similar fluorescent 2′-deoxyuridine derivatives that exhibit solvent-dependent photophysical properties via drastic changes in emission intensity as well as emission wavelength. These microenvironment-sensitive nucleosides may be used as probes for investigating nucleic acid dynamics and the recognition process. A deeper understanding of how the photophysical properties relate to chemical structures may allow for the design of ideal environmentally sensitive fluorescent nucleosides towards the development of DNA probes. Efforts in these directions are currently in progress.

## Supplementary Materials

Supplementary materials can be accessed at: http://www.mdpi.com/1420-3049/17/10/12061/s1.

## References

[1] Ranasinghe R.T., Brown T. (2005). Fluorescence based strategies for genetic analysis.. Chem. Commun..

[2] Wilson J.N., Kool E.T. (2006). Fluorescent DNA base replacements: Reporters and sensors for biological systems.. Org. Biomol. Chem..

[3] Venkatesan N., Seo Y.J., Bang E.K., Park S.M., Lee Y.S., Kim B.H. (2006). Chemical modification of nucleic acids toward functional nucleic acid systems.. Bull. Korean Chem. Soc..

[4] Venkatesan N., Seo Y.J., Kim B.H. (2008). Quencher-free molecular beacons: A new strategy in fluorescence based nucleic acid analysis.. Chem. Soc. Rev..

[5] Dodd D.W., Hudson R.H.E. (2009). Intrinsically fluorescent base-discriminating nucleoside analogs. Mini-Rev. Org. Chem..

[6] Sinkeldam R.W., Greco N.J., Tor Y. (2010). Fluorescent analogs of biomolecular building blocks: Design, properties, and application.. Chem. Rev..

[7] Dai N., Kool E.T. (2011). Fluorescent DNA-based enzyme sensors.. Chem. Soc. Rev..

[8] Østergaard M.E., Hrdlicka P.J. (2011). Pyrene-functionalized oligonucleotides and locked nucleic acids (LNAs): Tools for fundamental research, diagnostics, and nanotechnolog.. Chem. Soc. Rev..

[9] Hwang G.T., Seo Y.J., Kim B.H. (2004). A highly discriminating quencher-free molecular beacon for probing DNA.. J. Am. Chem. Soc..

[10] Ryu J.H., Seo Y.J., Hwang G.T., Lee J.Y., Kim B.H. (2007). Triad base pairs containing fluorene unit for quencher-free SNP typing.. Tetrahedron.

[11] Ryu J.H., Heo J.Y., Bang E.-K., Hwang G.T., Kim B.H. (2012). Quencher-free linear beacon systems containing 2-ethynylfluorenone-labeled 2′-deoxyuridine units.. Tetrahedron.

[12] Sinkeldam R.W., Greco N.J., Tor Y. (2008). Polarity of major grooves explored by using an isosteric emissive nucleoside.. ChemBioChem.

[13] Østergaard M.E., Guenther D.C., Kumar P., Baral B., Deobald L., Paszczynski A.J., Sharma P.K., Hrdlicka P.J. (2010). Pyrene-functionalized triazole-linked 2′-deoxyuridines-probes for discrimination of single nucleotide polymorphisms (SNPs).. Chem. Commun..

[14] Varghese R., Wagenknecht H.-A. (2010). Non-covalent versus covalent control of self-assembly and chirality of Nile red-modified nucleoside and DNA.. Chem. Eur. J..

[15] Bag S.S., Kundu R., Matsumoto K., Saito Y., Saito I. (2010). Singly and doubly labeled base-discriminating fluorescent oligonucleotide probes containing oxo-pyrene chromophore.. Bioorg. Med. Chem. Lett..

[16] Pawar M.G., Srivatsan S.G. (2011). Synthesis, photophysical characterization, and enzymatic incorporation of a microenvironment-sensitive fluorescent uridine analog. Org. Lett..

[17] Saito Y., Miyamoto S., Suzuki A., Matsumoto K., Ishihara T., Saito I. (2012). Fluorescent nucleosides with “on-off”switching function, pH-responsive fluorescent uridine derivatives.. Bioorg. Med. Chem. Lett..

[18] Tanaka M., Oguma K., Saito Y., Saito I. (2012). Enhancement of fluorescence quenching and exciplex formation in DNA major groove by double incorporation of modified fluorescent deoxyuridines. Bioorg. Med. Chem. Lett..

[19] Barrois S., Beyer C., Wagenknecht H.-A. (2012). Covalent modification of 2′-deoxyuridine with two different molecular switches.. Synlett.

[20] Tanaka M., Oguma K., Saito Y., Saito I. (2012). Drastic enhancement of excess electron-transfer efficiency through DNA by inserting consecutive 5-phenylethynyl-2'-deoxyuridines as a modulator.. Chem. Commun..

[21] Parker C.A. (1968). Photoluminescence of Solutions.

[22] Saito Y., Koda M., Shinohara Y., Saito I. (2011). Synthesis and photophysical properties of 8-arylbutadienyl 2′-deoxyguanosines.. Tetrahedron Lett..

[23] Saito Y., Shinohara Y., Ishioroshi S., Suzuki A., Tanaka M., Saito I. (2011). Synthesis of environmentally sensitive 2′-deoxyguanosine containing solvatochromic pyrene dluorophore.. Tetrahedron Lett..

[24] Panozzo S., Vial J.-C., Kervella Y., Stéphan O. (2002). Fluorene-fluorenone copolymer: Stable and efficient yellow-emitting material for electroluminescent devices.. J. Appl. Phys..

[25] Józefowicz M. (2007). Determination of reorganization energy of fluorenone and 4-hydroxyfluorenone in neat and binary solvent mixtures.. Spectrochim. Acta A.

[26] Nguyen D.D., Trunk J., Nakhimovsky L., Spanget-Larsen J. (2010). Electronic transitions of fluorene, dibenzofuran, carbazole, and dibenzothiophene: From the onset of absorption to the ionization threshold. J. Mol. Spectrosc..

[27] Sonogashira K., Tohda Y., Hagihara N. (1975). A convenient synthesis of acetylenes: Catalytic substitutions of acetylenic hydrogen with bromoalkenes, iodoarenes and bromopyridines.. Tetrahedron Lett..

[28] Hwang G.T., Son H.S., Ku J.K., Kim B.H. (2003). Synthesis and photophysical studies of bis-enediynes as tunable fluorophores.. J. Am. Chem. Soc..

[29] Sekine C., Ishitobi M., Iwakura K., Minai M., Fujisawa K. (2002). Novel high birefringence dibenzothiophenylacetylene liquid crystals.. Liq. Cryst..

[30] Lakowicz J.R. (2006). Principles of Fluorescence Spectroscopy.

[31] Eastman J.W. (1967). Quantitative spectrofluorimetry—The fluorescence quantum yield of quinine sulfate.. Photochem. Photobiol..

[32] Reichardt C. (1994). Solvatochromic dyes as solvent polarity indicators.. Chem. Rev..

[33] Sinkeldam R.W., Marcus P., Uchenik D., Tor Y. (2011). Multisensing emissive pyrimidine.. Chem. Phys. Chem..

[34] Sinkeldam R.W., Tor Y. (2007). To D or not to D? On estimating the microenvironment polarity of biomolecular cavities.. Org. Biomol. Chem..

[35] Cullinane N.M., Padfield H.J.H. (1935). Investigations in the diphenylene oxide series. Part V.. J. Chem. Soc..

