# Supplementary material for: Synthesis and Photophysical Study of 2′-Deoxyuridines Labeled with Fluorene Derivatives
Source: Molecules. 2012 Oct 15;17(10):12061–71. doi: 10.3390/molecules171012061 (PMC6268453; doi:10.3390/molecules171012061)

Table S1. Photophysical characteristics of nucleosides in water/1,4-dioxane binary solvent mixture.

Compound	Fraction water in 1,4-dioxane	$E_T(30)$ [a]	λ_{max} (nm) [b]	λ_{em} (nm) [c]	Stokes shift (cm^{-1})
U^{FL}	0	36.4	373	434	3768
	6.00E-04	36.6	373	434	3768
	0.003	37.3	373	434	3768
	0.015	39.9	373	434	3768
	0.042	43.4	373	434	3768
	0.06	44.7	373	434	3768
	0.12	46.8	373	435	3821
	0.21	48.6	374	436	3802
	0.42	52.1	374	444	4215
	0.6	54.9	383	454	4083
U^{FO}	0	36.4	344	516	9690
	6.00E-04	36.6	345	519	9718
	0.003	37.3	346	518	9597
	0.015	39.9	346	529	9998
	0.042	43.4	345	535	10294
	0.06	44.7	346	543	10486
	0.12	46.8	345	546	10670
	0.21	48.6	346	544	10519
	0.42	52.1	343	553	11071
	0.6	54.9	344	551	10921
U^{DBF}	0	36.4	328	384	4446
	6.00E-04	36.6	328	384	4446
	0.003	37.3	328	384	4446
	0.015	39.9	328	385	4514
	0.042	43.4	328	386	4581
	0.06	44.7	329	386	4488
	0.12	46.8	328	388	4715
	0.21	48.6	329	389	4688
	0.42	52.1	328	399	5425
	0.6	54.9	328	414	6333
U^{DBT}	0	36.4	327	383	4471
	6.00E-04	36.6	327	383	4471
	0.003	37.3	327	383	4471
	0.015	39.9	327	383	4471
	0.042	43.4	327	383	4471
	0.06	44.7	327	385	4607
	0.12	46.8	327	387	4741
	0.21	48.6	327	389	4874
	0.42	52.1	327	397	5392
	0.6	54.9	327	412	6309

[a] $E_T(30)$ values are taken from [Sinkeldam, R. W.; Tor, Y. *Org. Biomol. Chem.* **2007**, *5*, 2523–2528];
[b] Only the largest absorption maxima are listed; [c] Wavelength of emission maximum when excited at the absorption maximum.

Figure S1. ^{1}H-NMR spectra of (**a**) **U^{DBF}** and (**b**) **U^{DBT}**.

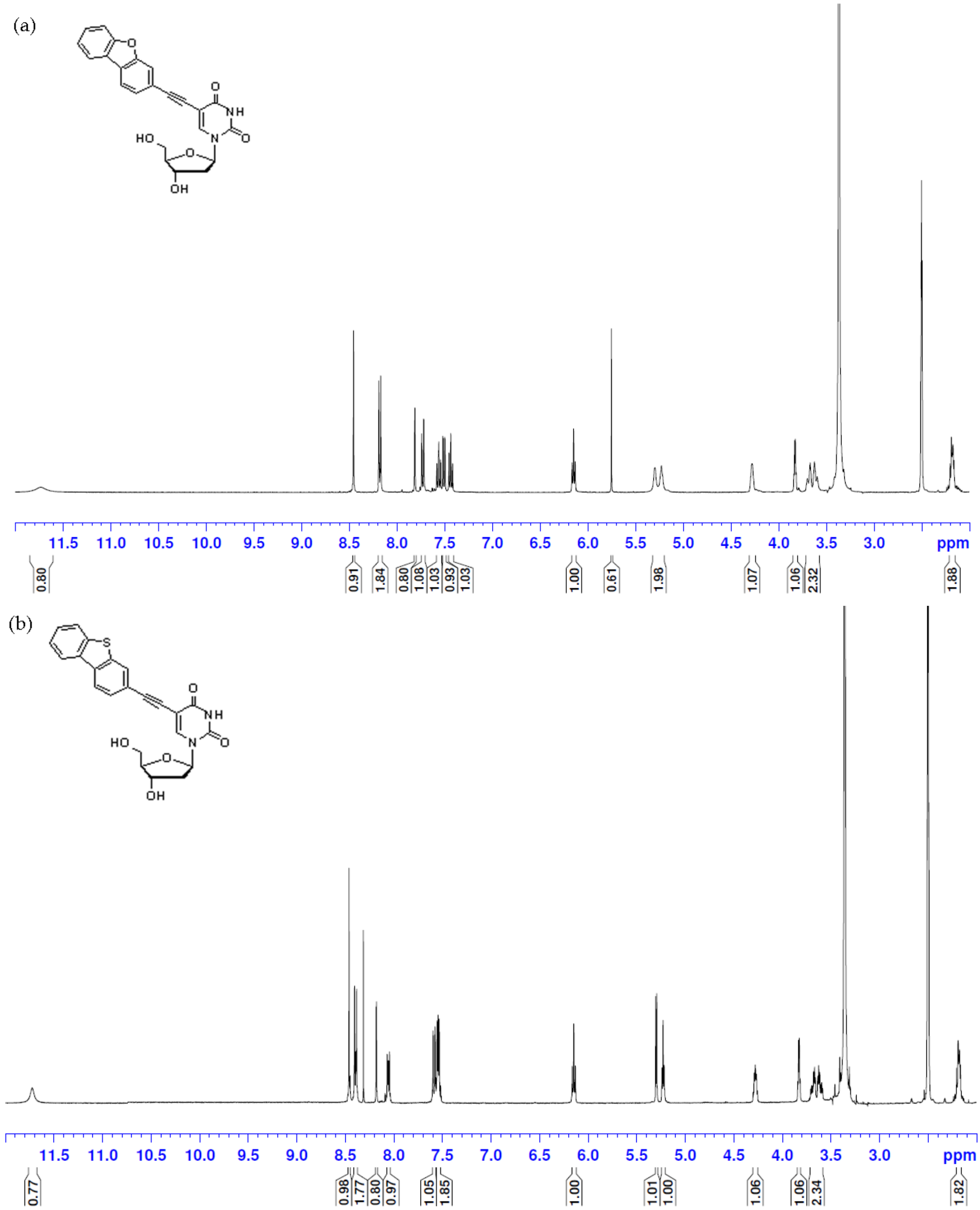

Supplement: Supplementary file 1 [file molecules-17-12061-s001.pdf]
